# Negative optical force field on supercavitating titanium nitride nanoparticles by a single plane wave

**DOI:** 10.1515/nanoph-2021-0503

**Published:** 2021-11-10

**Authors:** Eungkyu Lee, Tengfei Luo

**Affiliations:** Department of Electronic Engineering, Kyung Hee University, Yongin-si 17104, Gyeonggi-do, Republic of Korea; Department of Aerospace and Mechanical Engineering, University of Notre Dame, Notre Dame 46556, IN, USA; Department of Chemical and Biomolecular Engineering, University of Notre Dame, Notre Dame 46556, IN, USA; Center for Sustainable Energy of Notre Dame (ND Energy), University of Notre Dame, Notre Dame 46556, IN, USA

**Keywords:** optical pulling, plasmononics, single plane wave, supercavitation, TiN nanoparticle

## Abstract

A pulling motion of supercavitating plasmonic nanoparticle (NP) by a single plane wave has received attention for the fundamental physics and potential applications in various fields (*e.g.*, bio-applications, nanofabrication, and nanorobotics). Here, the supercavitating NP depicts a state where a nanobubble encapsulates the NP, which can be formed via the photo-thermal heating process in a liquid. In this letter, we theoretically study the optical force on a supercavitating titanium nitride (TiN) NP by a single plane wave at near-infrared wavelengths to explore optical conditions that can potentially initiate the backward motion of the NP against the wave-propagating direction. An analysis with vector spherical harmonics is used to quantify the optical force on the NP efficiently. Next, the vector field line of the optical force is introduced to visualize the light-driven motion of the NP in a nanobubble. Finally, we characterize the vector field lines at various optical conditions (*e.g.*, various sizes of NP and nanobubble, and wavelength), and we find a suitable window of the optical state which can potentially activate the backward motion of the supercavitating TiN NP.

Optical manipulation of nanoparticles (NPs) in fluids has drawn significant interest as it can be leveraged in various fields such as biological applications (*e.g.*, molecular sensing [1, 2], and drug delivery [3, 4]) and nanotechnologies (*e.g.*, nanopatterning [5], [6], [7], [8], and nanorobotics [9], [10], [11]). The optical force, which arises from the momentum exchange of propagating photons with the NP, is vital to guiding the NP [12], [13], [14], [15], [16], [17], [18]. Recently, it has been experimentally demonstrated that a loosely focused Gaussian beam (*i.e.*, a single plane-like wave) can pull a plasmonic Au NP in the water against the beam-propagating direction for a distance of ∼0.1 cm, which is the largest travel distance of light-pulled NP in a solution [19], [20], [21], [22]. Since this finding, manipulating an NP with a single plane wave [23], [24], [25], [26] has received more attention not only for fundamental interest but also for its potential applications. Usually, a single plane wave can apply an optical force on an NP in the wave-propagating direction, which can only push the NP to move in the forward direction [27], [28], [29]. The pulling of NP with a single plane wave seems to be forbidden by the momentum conservation rule. From the practical point of view, the backward motion of NP can offer an additional degree of freedom in optical manipulation [10, 27]. Furthermore, as the plane wave can be easily achieved with loosely focused Gaussian beam without complex active/passive modulators, it has ubiquitous accessibility.

The mechanism of the observed backward motion of the Au NP has been understood with the so-called ‘negative optical force’ when the NP is encapsulated by a plasmonic nanobubble (*i.e.*, supercavitating NP) [19, 21]. Here, the negative optical force means that the direction of the optical force along the optical axis is opposite to the beam-propagating direction [16, 23], [24], [25, 28, 30, 31]. The plasmonic Au NP can form a nanobubble when it supports a strong photo-thermal energy conversion process by efficiently absorbing the incident light [32], [33], [34]. The negative optical force is originated from the optical condition that the refractive index of nanobubble is lower than that of water [21]. In this condition, the nanobubble/water interface can act as an optical mirror to redirect the photon stream opposite to the beam-propagating direction [19]. An electromagnetic multipole analysis has also shown that the negative optical force can be from unique electric dipole–quadrupole and electric dipole–magnetic dipole interactions in a nanobubble. Quantitatively, the force components from these interactions can be negative when the spherical cavity has a lower refractive index than the medium [21]. As a result, the backward photon stream can apply negative radiative pressure to the NP in the nanobubble, driving the NP to contact the interface at the light-incoming side of the nanobubble. At the same time, as the NP can keep the high temperature (>550 K) under the continuous irradiation of the laser beam, it can instantly evaporate water molecules at the contacted interface like the Leidenfrost effect [35]. This nanoscale effect allows the NP to extend the boundary of the nanobubble toward the backward direction, leading to a high-speed motion.

In the meantime, the negative optical force on the supercavitating NP can be more obvious when the wavelength (*λ*) of the incident plane wave moves to a longer *λ* away from the surface plasmonic resonance (SPR) peak of the NP [21]. It is likely that in the off-SPR region, the lower-index cavity effect of nanobubble (*i.e.*, the characteristics of internal field profile in the nanobubble) on the optical force can be more dominated as the resonance effect of the NP is diminished. In practice, this can offer additional advantages, especially for bio-applications leveraging the backward motion of plasmonic NP. For example, in bio-applications, if one needs to optically grab (*i.e.*, optical tweezer) a functionalized metallic NP located deep in a sample, the optical aberration effect [36] can compromise the tweezing strength on the NP. In this case, optically pulling the NP to the surface of the sample can be very helpful. At the same time, as the SPR peak of plasmonic NPs can be tuned in 400 nm < *λ* < 800 nm [37], the off-SPR region can be in the near-infrared (NIR) region of 800 nm < *λ* < 1200 nm, which is in the biologically transparent window [10]. Thus, it can minimize undesired interactions between biomolecules and the light source when optically pulling a metallic NP in a sample. Moreover, manipulating a metallic NP near the sample surface can minimize undesired volumetric heating [20], as the focal point of the beam does not need to be placed deep in the sample.

However, the absorption quality factor $\left(Q_{a}\right)$ of Au NP drops quickly when the wavelength moves away from the SPR peak (*i.e.*, the bandwidth of absorption is narrow) [37]. It implies that at the off-SPR region, the absorption quality factor can be infinitesimal, and thus forming a nanobubble with the NPs may require high intensity light that is not a suitable in practical applications [20]. In addition, when the Au NP is supercavitated, several studies have reported that Au compounds are easy to deteriorate due to the low melting temperature (*e.g.*, reshaped or fused with other NPs [38]), limiting the recycling of these NPs in applications. Therefore, for bio-applications that may benefit from the backward motion of supercavitating NP, it is necessary to explore other materials other than Au NPs.

In this letter, we theoretically explore the optical force on a TiN NP in a nanobubble to examine the possibility of backward motion driven by a single plane wave. TiN NPs are bio-compatible and have a broad wide bandwidth of nontrivial *Q*_a_ extending to the biologically transparent window and a melting temperature of ∼3000 K [39], which is 2.4 times higher than that of Au. We use vector spherical harmonics to efficiently estimate the optical force and introduce the vector field lines of optical force, which can effectively visualize the light-driven dynamics of the NP in a nanobubble. The characteristics of vector field lines are investigated for various optical configurations (*e.g.*, wavelength, the size of nanobubble, and NP), and we find that there is a suitable geometrical window to realize the backward motion of TiN NP. For example, at *λ* = 1050 nm, the nanobubble with a radius between 120–200 nm can lead to negative optical forces on a TiN NP with a radius of 35 nm. The negative optical force can bring the NP to the nanobubble surface facing the incident plane wave.

In the supercavitating NP phenomenon, this study focuses on the intermediate regime after nanobubble formation and before the activation of the ballistic motion. Specifically, we are interested in discovering optical conditions (*λ*, the size of nanobubble and NP) to induce the negative optical force when an off-SPR, near-infrared (NIR) plane wave is incident to a supercavitating TiN NP. We consider a solid spherical TiN NP encapsulated by a nanobubble as a representative case in this study (see Figure 1 for the geometrical configuration). The optical force (F) on the TiN NP can be estimated by integrating the flux of Maxwell’s stress tensor over a surface enclosing the NP [40]. To efficiently perform the integration, we use the analytical form of optical force on a spherical NP at a given location (r) in a nanobubble, where F can be expressed with the complex coefficients of vector spherical harmonics in the incident and scattering light-fields of the NP (see Supplementary Information for the details). Also, for simplicity, we use the 1st order scattering approximation, which is valid at the wavelength region far from the SPR peak [21]. This study focuses on a low fluence range (<∼50 mJ/cm^2^), where the ballistic motion of supercavitating NP has been experimentally observed [19, 21]. In this fluence range, experimental studies [34] have shown that the radius (*R*_b_) of plasmonic nanobubble can be 100 nm $\leq R_{\text{b}}\leq$ 250 nm, which is the range we study.

**Figure 1:**
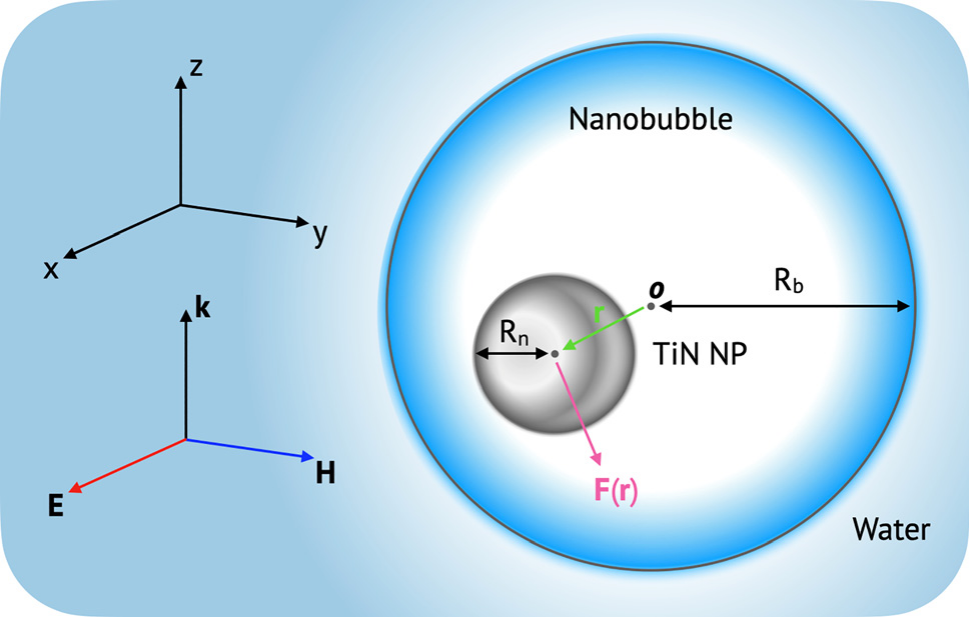
Schematic of optical configuration of a TiN NP in a nanobubble, which is in the medium of water. **F**(**r**) is the optical force on the TiN NP, where **r** is the center of NP. **k**, **E**, and **H** denote the wave vector, the electric field, and the magnetic field of an incident plane wave, respectively. $R_{\text{n}}$ is the radius of TiN NP; $R_{\text{b}}$ is the radius of nanobubble. ***o*** is the origin of the Cartesian coordinate system.

To quickly evaluate optical conditions enabling the negative optical force, the z-component of optical force $\left({\text{F}}_{\text{z}}\right)$ on the NP is investigated as a function of λ and the radius of NP $\left(R_{\text{n}}\right)\text{.}$ It is noted that the negative optical force means that the sign of ${\text{F}}_{\text{z}}$ is negative. We assume that the incident plane wave is *x*-polarized and propagates to the positive *z*-direction; the NP is encapsulated by a nanobubble with *R*_b_ = 150 nm, and it is located at **r** = 0, 0, −75 nm where the center of the nanobubble is the origin; the refractive index of nanobubble and water are, respectively, 1 and 1.33; the complex optical index of TiN (*n* = 1.7–3.0, *k* = 3.6–5.7) is from reference [41]. In Figure 2, we can see that the negative optical force appears in the NIR region of 800 nm ≤λ≤ 1500 nm for the NP with the size of 10 nm $\leq R_{\text{n}}\leq$ 45 nm. As the TiN NP has the SPR peak between 500 nm ≤λ≤ 700 nm [39], the region of 800 nm ≤λ≤ 1500 nm corresponds to the off-SPR region. This characteristic corresponds well to the observed trend in various NPs such as Au, Si, or SiO_2_ NP, when they are in nanobubble [21].

**Figure 2:**
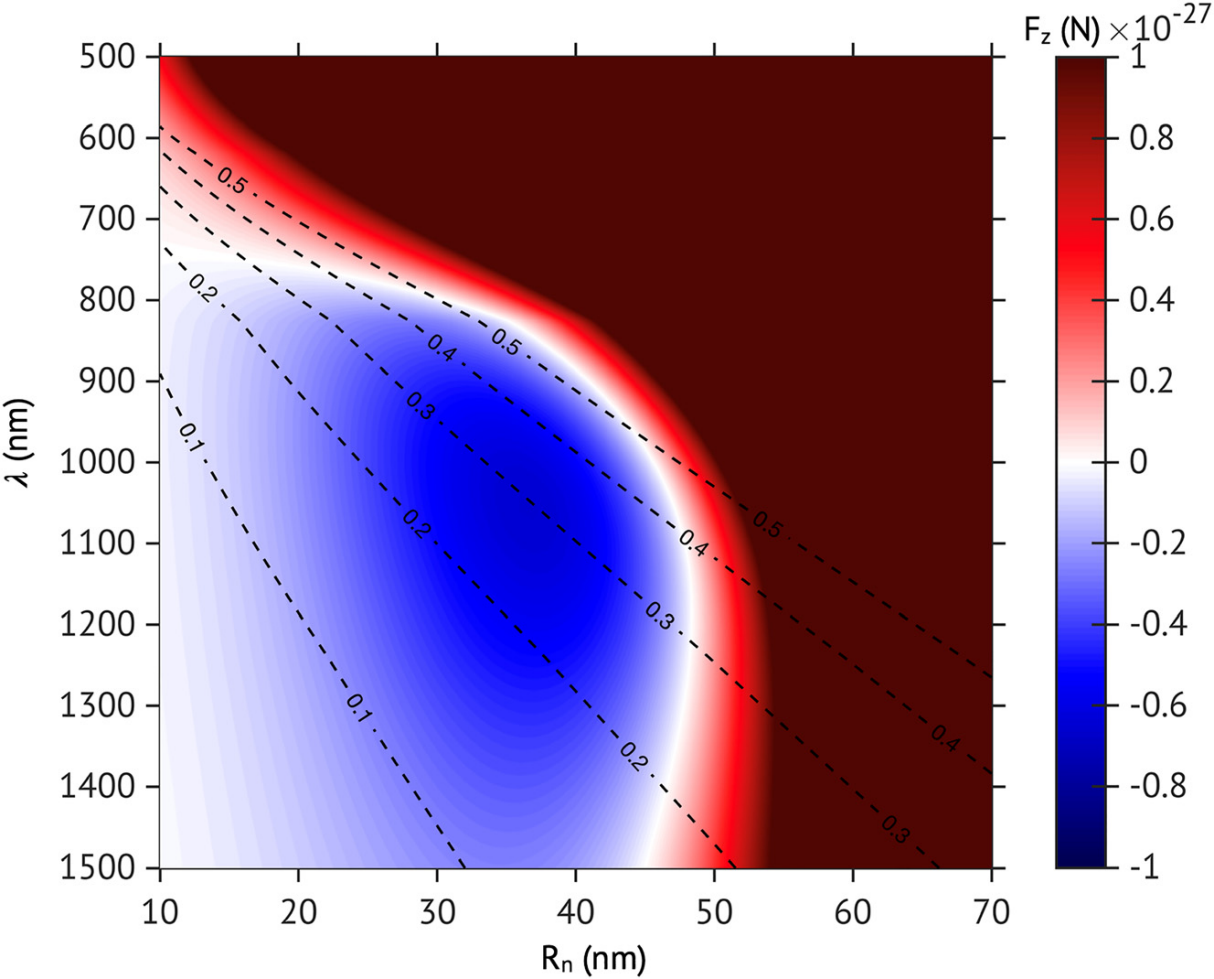
The calculated z-component of optical force on a TiN NP in a nanobubble as a function of the wavelength (*λ*) of the incident plane wave and *R*_n_. Here, *R*_b_ = 150 nm, **r** = 0, 0, −75 nm, and the amplitude of incident electric field is unity (*i.e.*, |E| = 1). The dashed lines show the calculated absorption quality factor (*Q*_a_) when the NP is immersed in water without a nanobubble.

At the same time, we also investigate the optical absorption properties of the TiN NP in the off-SPR region, and we plot the contour lines of optical absorption quality factor (*Q*_a_) in Figure 2 as well. The optical absorption is vital to forming a nanobubble as it is the factor that the photo-thermal heating rate is proportional to [32]. The temperature of NP should reach a threshold (*e.g.*, the spinodal temperature of the water, 550 K) to be encapsulated by a nanobubble [32]. For estimating the quality factor, we use the Mie theory with the complex optical index of TiN in reference [41] (see Supplementary Information for the details) and assume that a TiN NP is immersed in water. In the wavelength region where the negative optical force appears, the TiN NP has an absorption quality factor of 0.1∼0.5, which is at a promising level to form a photo-thermal nanobubble in experiments. For example, a TiN NP with *R*_n_ = 35 nm in the water can reach a temperature of 500–1000 K by a femtosecond pulsed laser with a fluence (*F*) of 7–24 mJ/cm^2^ at *λ* = 1050 nm. These values are comparable to the threshold fluences of silica-core Au–shell NP in water at the SPR peak, where the core–shell NP has exhibited the supercavitating motion in experiments [19, 34]. Also, at the infrared region (800 nm ≤λ≤ 1500 nm), the absorption quality factor of TiN NP (*Q*_A_ = 0.1) is one order of magnitude higher than that of solid Au NP with the radius of 10–70 nm (*Q*_A_ of solid Au NP ∼ 0.01, see Supplementary Information Section 4). We noted that the following equation is used to roughly estimate the threshold fluence for the TiN NP: $F=\left(T_{\text{n}}-T_{\text{RT}}\right)C_{\text{TiN}}V_{\text{n}}/A_{\text{n}}Q_{\text{A}}$ , where $C_{\text{TiN}}$ is the volumetric heat capacity of TiN, $T_{\text{RT}}$ is the room temperature, $V_{\text{n}}$ is the volume of NP, $A_{\text{n}}$ is the cross-sectional area of NP; $C_{\text{TiN}}$ = 2.0209 × 10^6^ J/m^3^K, $T_{\text{RT}}$ = 300 K, $Q_{\text{A}}$ = 0.28.

In the optical condition showing the negative optical force, we choose a state of $R_{\text{n}}$ = 35 nm and λ = 1050 nm to examine the light-driven dynamics of NP in the nanobubble. To effectively visualize the distribution of F , the vector field lines (S) of optical force is considered, and it is defined with the recursion relation:

(1) $$\begin{array}{l} \text{S}=\left\{S_{i}|S_{i}=\left[x_{i}\text{, }y_{i}\text{, }z_{i}\right],\;i\in \mathbb{Z}\right\}\text{,}\\ x_{i\pm 1}=x_{i}\pm \delta F\left(x_{i},y_{i},z_{i}\right)\cdot \hat{x}\text{,}\\ y_{i\pm 1}=y_{i}\pm \delta F\left(x_{i},y_{i},z_{i}\right)\cdot \hat{y}\text{,}\\ z_{i\pm 1}=z_{i}\pm \delta F\left(x_{i},y_{i},z_{i}\right)\cdot \hat{z}\text{.} \end{array}$$

To finely track points ( $S_{i}$ ) in a vector field line, we tune δ to keep |*S*_*i*+1_ − *S*_*i*_| ≪ *R*_n_, where δ is a positive and has a unit of m/N. This condition ensures that the distance between the two nearest neighboring points in a vector field line is much smaller than the size of the NP.

In experiments, upon the illumination of light, the plasmonic NP can get encapsulated by a nanobubble in 1–100 ns (*i.e.*, supercavitating process) by the photo-thermal energy conversion process [33]. In the NP-water suspension, the evolution of nanobubble is a thermodynamically nonequilibrium process involving multiple phases, posing a theoretical difficulty to track the motion of NP in expanding nanobubbles. In this study, without the loss of generality, the vector field line can represent the motion of TiN NP in a nanobubble given following assumption: the NP is instantly encapsulated by a nanobubble of a certain size when it is illuminated by a light source, and then the optical force drives the NP to move in the nanobubble until the NP reaches the nanobubble interface. This assumption is valid since the magnitude of the optical force in real experiments would be 10^−12^ N–10^−14^ N [19, 21], which drives the NP to move 0.01–1 nm during the supercavitating process, which lasts 1–100 ns: Stoke’s law considering the steam viscosity (10^−5^ kg/m s) yields the speed of NP of 10^−2^ m/s for the optical forces, which corresponds to the travel distance of 0.01–1 nm during the bubble-forming time of 1–100 ns. It is also noted that the motion of NP would be limited by the viscous force of steam in the nanobubble, as the motion of NP is at a low Reynold number of ∼10^−3^ ≪ 1.

We calculate the vector field line in Eq. (1) by randomly seeding fifty spots of *S*_0_ for the nanobubble with various *R*_b_ = 100 nm, 150 nm, 200 nm, and 250 nm, and plot them in Figure 3. With an assigned *S*_0_, a vector field line can be completed by taking the plus (or minus) sign in Eq. (1). On a vector field line, the color corresponds to the normalized z-component of the optical force at a given location, and it can guide us to know the moving direction of NP visually. For example, for the case of *R*_b_ = 100 nm, the vector field line denoted as ‘**A**’ can bring the TiN NP to the light-outgoing interface of nanobubble (the color is close to red, showing that ${\text{F}}_{\text{z}}$ is positive), potentially activating the forward motion of supercavitating NP. In the meantime, when the NP get encapsulated by a nanobubble, the probability that the NP reaches to the certain side of interface (*e.g.*, top or bottom side of nanobubble) may depend on the number density of associated vector field lines. In the nanobubble of *R*_b_ = 100 nm, most of the vector field lines observed in Figure 3a can be categorized into a kind of ‘**A**’. Thus, it is most likely that this nanobubble size will initiate the forward motion, and it may be difficult to activate the backward motion.

**Figure 3:**
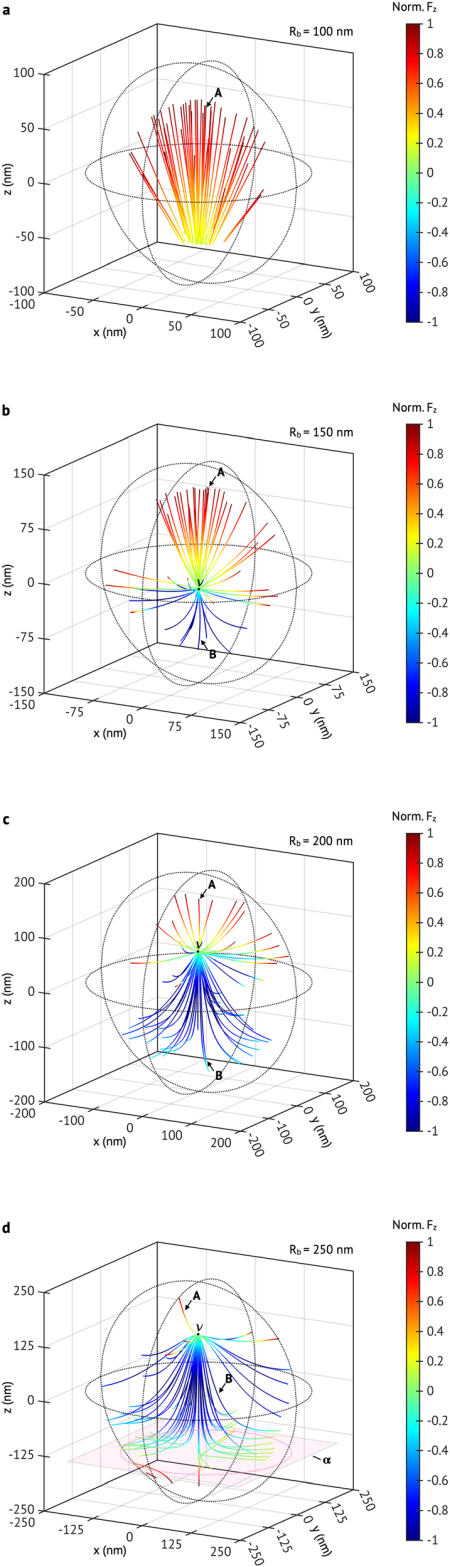
The vector field lines of optical force on a TiN NP in various sizes of nanobubbles: (a) *R*_b_ = 100 nm (b) *R*_b_ = 150 nm, (c) *R*_b_ = 200 nm (d) *R*_b_ = 250 nm. In (a) to (d), *R*_n_ = 35 nm, and *λ* = 1050 nm; the color of each vector field line corresponds to the normalized z-component of optical force, which is leveled by the color bar. In (a)–(d), **A** (or **B**) denotes the representative vector field line which can guide the TiN NP to the top (or bottom) side of nanobubble; ν depicts a nodal point where the two kinds (**A** and **B**) of vector field lines are emerged out; α depicts an asymptotic plane where vector field lines cannot pass, but it lets the vector field lines go to the rim on the nanobubble surface (the magenta-dotted circle).

Meanwhile, a nodal point denoted as ‘ ν ’ appears for the case of *R*_b_ = 150 nm, as shown in Figure 3b. Below the nodal point (z < $\nu_{\text{z}}$ ), a new kind of vector field line denoted as ‘**B**’ emerges. This vector field line is in blue color, indicating the negative sign of ${\text{F}}_{\text{z}}$ , and the blue color is extended to the light-incoming interface of the nanobubble. Thus, the B-type vector field line can guide the NP to move to the light-incoming interface. If the NP is on the B-type vector field line, the backward ballistic motion can be potentially enabled. At the same time, we can still see that there are many **A**-type vector field lines in the top part of the nanobubble (above the nodal point), and the number of the **A**-type field lines is greater than that of the **B**-type ones. However, for the nanobubble of *R*_b_ = 200 nm, the situation is reversed. It seems that the nodal point ‘ ν ’ moves up to the positive *z*-direction, and the **B**-type vector field lines are more frequently seen than the **A**-type ones (see Figure 3c). Therefore, with the nanobubble of *R*_b_ = 150–200 nm, it is expected that both the forward and backward ballistic motion can be initiated, but the probability of observing the backward motion may be higher if the radius of nanobubble is close to 200 nm.

In the nanobubble of *R*_b_ = 250 nm, we find that an asymptotic plane (indicated by ‘ α ’ in Figure 3d) emerges near the bottom part of the nanobubble. The **B**-type vector field lines from the nodal point cannot reach the light-incoming interface of the nanobubble due to the asymptotic plane, and they extend toward the rim of the nanobubble (see the magenta-dotted circle in Figure 3d). Similarly, a few red-colored vector field lines are seen below the asymptotic plane, and they also cannot pass the plane but approaches the rim on the nanobubble surface. Therefore, the TiN NP on such vector field lines can be pushed toward the rim of the nanobubble. Accordingly, we may expect that the ballistic motion initiated by these vector lines largely deviates from the optical axis, and the backward motion would not be observed as no vector field lines can guide the NP to the bottom interfaces of nanobubble.

From the above results, we assume that there is an ideal range of nanobubble size, where the backward motion of supercavitating NP can be initiated (see Figure 4). The range may be defined as $R_{\nu }\leq R_{\text{b}}\leq R_{\alpha }$ , where $R_{\nu }$ (or $R_{\alpha }$ ) denotes the minimum radius of nanobubble where the nodal point (or the asymptotic plane) is observed. Figure 4 shows the representative case of the range for various sizes of TiN NP at *λ* = 1050 nm, where the range that can enable backward motion is highlighted in gray. The zone indicates that the radius of NP should be smaller than 50 nm to initiate the backward motion, and smaller NPs would have wider windows in *R*_b_. For example, a TiN NP of *R*_n_ = 15 nm can have backward motion if 100 nm $\leq R_{\text{b}}\leq$ 210 nm, while an NP of *R*_n_ = 45 nm will need 140 nm $\leq R_{\text{b}}\leq$ 185 nm to achieve backward motion. However, since smaller NP has low optical absorption efficiency than larger NP (see Figure 2), real experiments may need to use higher intensity light to leverage the wider *R*_b_ range. We note that a nanobubble of *R*_b_ = 100 nm is assumed to be the minimum radius that the NP can form in water, but it does not compromise the physics inferred from this study. Also, if the NP size is bigger than *R*_n_ = 50 nm, no backward motion is achievable disregard the nanobubble size, as the $R_{\nu }$ overlaps with $R_{\alpha }$ .

**Figure 4:**
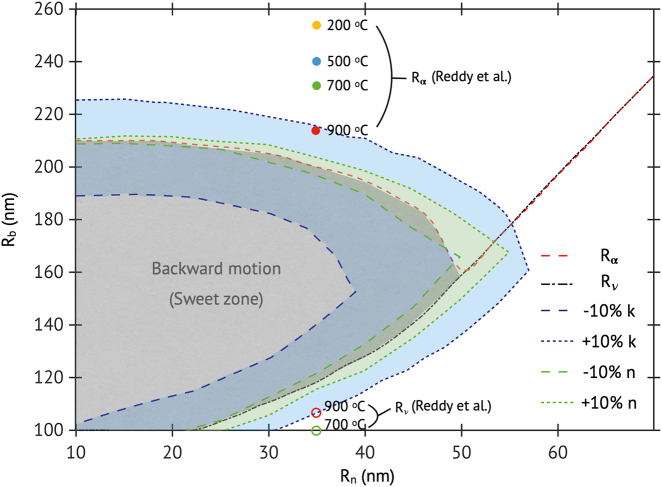
The minimum radius of nanobubble where the nodal point $\left(R_{\nu }\right)$ or the asymptotic plane $\left(R_{\alpha }\right)$ is observed. The region highlighted in gray, which is estimated with the refractive index from [41], indicates the conditions that can potentially initiate the backward ballistic motion of TiN NPs. The blue and green dash (or dot) lines, respectively, indicate the boundary of the sweet zone when the imaginary and the real part of refractive index increase (or decreases) by 10%. The circles indicate the calculated $\left(R_{\alpha }\right)$ (filled) and $R_{\nu }$ (opened) of a 35-nm-radius TiN NP with the temperature-dependent refractive index from Reddy et al. [42].

In the meantime, it has been reported that the complex refractive index of TiN depends on factors such as temperature [42], or fabricating conditions [43]. We investigate the effect of refractive index variation on the sweet zone where the refractive index taken from reference [40] at *λ* = 1050 nm is increased (or decreased) by 10%. It finds that the sweet zone is more sensitive to the change of the imaginary part (*k*) of the refractive index than that of the real part (*n*) (see Figure 4). The area of the sweet zone can be enlarged (or reduced) when the magnitude of *n* and *k* increases (or decreases). However, it is noted that the change of refractive index will not influence the physics inferred from our analysis. In addition, Reddy et al. [42] have reported the variation of the refractive index of TiN thin film with the change of temperature (referred to as Reddy’s index). The temperature-dependent refractive index can be important to the supercavitating TiN NP, as it is based on the photo-thermal energy conversion process. We thus investigate $R_{\nu }$ (or $R_{\alpha }$ ) with Reddy’s index for the case of *λ* = 1050 nm and $R_{\text{n}}$ = 35 nm. In Figure 4, it is seen that as the temperature increases to 900 °C from 200 °C, $R_{\alpha }$ (or $R_{\nu }$ ) moves to the smaller (the larger) radius, possibly leading to the reduction of the sweet zone size. We also estimated the sweet zone of solid Au NP at *λ* = 1050 nm for a comparison (see Supplementary Information Section 4) and found that the solid Au NP usually has a larger sweet zone than that of the TiN NP with the same size. This is due to the imaginary part of the refractive index of Au being higher than that of TiN. However, as discussed above, the Au NP needs a much higher intensity of infrared light to be supercavitated due to the inferior absorption quality factor.

In conclusion, we study the optical force field on a TiN NP when the NP is in a nanobubble to investigate the possibility of backward motion enabled by a single plane wave at the off-SPR wavelength. Within a nanobubble, a TiN NP with a size smaller than *R*_n_ = 50 nm can receive a negative optical force, which is needed to achieve backward motion. At the off-SPR region of 800 nm < *λ* < 1500 nm, the TiN NP (*R*_n_ < 50 nm) shows the optical absorptions of 0.1 < *Q*_a_ < 0.5, which is at a promising level to form a nanobubble in experiment. The analysis of the vector field lines of the optical force finds that the nodal point and the asymptotic plane can emerge in a nanobubble, depending on its size. We use this point and this plane to find the range of nanobubble size that can potentially lead to the backward motion of the encapsulated NP. The results from this study may provide valuable information to experimentally realize optical pulling of TiN NPs for potential applications.

## Supplementary Material

Supplementary Material
