# Supplementary material for: Negative optical force field on supercavitating titanium nitride nanoparticles by a single plane wave
Source: Nanophotonics. 2021 Nov 10;11(1):79–86. doi: 10.1515/nanoph-2021-0503 (PMC11501752; doi:10.1515/nanoph-2021-0503)
Supplement: Supplementary file 1 — Supplementary Material [file j_nanoph-2021-0503_suppl.docx]

**1. Analytical form of optical force on a nanoparticle**

The incident ($\mathbf{E}_{\mathbf{i}}$) and scattered ($\mathbf{E}_{\mathbf{s}}$) electric field for a spherical shape of nanoparticle can be expressed with the vector spherical harmonics as:

$$\mathbf{E}_{\mathbf{i}}= \sum_{n=1}^{\infty} \sum_{m=-n}^{n} \left[ p_{nm}\boldsymbol{N}_{nm}^{\left( 1 \right)}+q_{nm}\boldsymbol{M}_{nm}^{\left( 1 \right)} \right],$$

$$\mathbf{E}_{\mathbf{s}}= \sum_{n=1}^{\infty} \sum_{m=-n}^{n} \left[ a_{nm}\boldsymbol{N}_{nm}^{\left( 3 \right)}+b_{nm}\boldsymbol{M}_{nm}^{\left( 3 \right)} \right],$$

$$\boldsymbol{N}_{nm}^{\left( j \right)}\boldsymbol{=}\frac{1}{k}\nabla\times\boldsymbol{M}_{nm}^{\left( j \right)},$$

$$\boldsymbol{M}_{nm}^{\left( j \right)}=\nabla\times\boldsymbol{r}u_{nm}^{\left( j \right)}\boldsymbol{,}$$

$$u_{nm}^{\left( 1 \right)}\boldsymbol{=}j_{n}\left( kr \right)P_{n}^{m}\left( \cos\theta\right)e^{im\phi},$$

$$u_{nm}^{(3)}\boldsymbol{=}\left( j_{n}\left( kr \right)+iy_{n}\left( kr \right) \right)P_{n}^{m}\left( \cos\theta\right)e^{im\phi}.$$

where $j_{n}$ and $y_{n}$ are spherical Bessel functions, and $P_{n}^{m}$ is associated Legendre function. Salandrino et al. [1] have shown that the time-averaged optical force ($\mathbf{F}$) on the nanosphere can be expressed with $p_{nm}$, $q_{nm}$, $a_{nm}$, and $b_{nm}$ as the following:

$$\mathbf{F}=\frac{\pi\epsilon_{0}}{k_{0}^{2}}\sum_{n=1}^{\infty} \sum_{m=0}^{n} \left[ F_{nm}^{x}\hat{\boldsymbol{x}}\boldsymbol{+}F_{nm}^{y}\hat{\boldsymbol{y}}\boldsymbol{+}F_{nm}^{z}\hat{\boldsymbol{z}} \right],$$

$$F_{nm}^{j}=F_{nm}^{j,ss}+{\left( \frac{1}{2} \right)F}_{nm}^{j,si}, \mathrm{where} j=x,y, \mathrm{or} z,$$

$$F_{nm}^{x,ss}=C_{nm}^{1}\left\{ \left( 1-\delta_{m,0} \right)\left[ \left( 1+\delta_{m,1} \right)\mathrm{Im}\left( \alpha_{enm}^{s}\alpha_{e\left( n+1 \right)\left( m-1 \right)}^{s*}+\beta_{enm}^{s}\beta_{e\left( n+1 \right)\left( m-1 \right)}^{s*} \right)+\left( 1-\delta_{m,1} \right)\mathrm{Im}\left( \alpha_{onm}^{s}\alpha_{o\left( n+1 \right)\left( m-1 \right)}^{s*}+\beta_{onm}^{s}\beta_{o\left( n+1 \right)\left( m-1 \right)}^{s*} \right) \right] \right\}-C_{nm}^{2}\left\{ \left( 1+\delta_{m,0} \right)\mathrm{Im}\left( \alpha_{enm}^{s}\alpha_{e\left( n+1 \right)\left( m+1 \right)}^{s*}+\beta_{enm}^{s}\beta_{e\left( n+1 \right)\left( m+1 \right)}^{s*} \right)+\left( 1-\delta_{m,0} \right)\mathrm{Im}\left( \alpha_{onm}^{s}\alpha_{o\left( n+1 \right)\left( m+1 \right)}^{s*}+\beta_{onm}^{s}\beta_{o\left( n+1 \right)\left( m+1 \right)}^{s*} \right) \right\}+C_{nm}^{3}\left\{ \left( 1+\delta_{m,0} \right)\mathrm{Im}\left( \alpha_{enm}^{s}\beta_{on(m+1)}^{s*} \right)+\left( 1-\delta_{m,0} \right)\mathrm{Im}\left( \alpha_{en(m+1)}^{s}\beta_{onm}^{s*} \right) \right\}+C_{nm}^{4}\left\{ \left( 1-\delta_{m,0} \right)\left[ \left( 1-\delta_{m,1} \right)\mathrm{Im}\left( \beta_{enm}^{s}\alpha_{on(m-1)}^{s*} \right)+\left( 1+\delta_{m,1} \right)\left( \beta_{en(m-1)}^{s}\alpha_{onm}^{s*} \right) \right] \right\},$$

$$F_{nm}^{x,si}=C_{nm}^{1}\left\{ \left( 1-\delta_{m,0} \right)\left[ \left( 1+\delta_{m,1} \right)\mathrm{Im}\left( \alpha_{enm}\alpha_{e\left( n+1 \right)\left( m-1 \right)}^{s*}+\alpha_{enm}^{s}\alpha_{e\left( n+1 \right)\left( m-1 \right)}^{*}+\beta_{enm}\beta_{e\left( n+1 \right)\left( m-1 \right)}^{s*}+\beta_{enm}^{s}\beta_{e\left( n+1 \right)\left( m-1 \right)}^{*} \right)+\left( 1-\delta_{m,1} \right)\mathrm{Im}\left( \alpha_{onm}\alpha_{o\left( n+1 \right)\left( m-1 \right)}^{s*}+\alpha_{onm}^{s}\alpha_{o\left( n+1 \right)\left( m-1 \right)}^{*}+\beta_{onm}\beta_{o\left( n+1 \right)\left( m-1 \right)}^{s*}+\beta_{onm}^{s}\beta_{o\left( n+1 \right)\left( m-1 \right)}^{*} \right) \right] \right\}-C_{nm}^{2}\left\{ \left( 1+\delta_{m,0} \right)\mathrm{Im}\left( \alpha_{enm}\alpha_{e\left( n+1 \right)\left( m+1 \right)}^{s*}+\alpha_{enm}^{s}\alpha_{e\left( n+1 \right)\left( m+1 \right)}^{*}+\beta_{enm}\beta_{e\left( n+1 \right)\left( m+1 \right)}^{s*}+\beta_{enm}^{s}\beta_{e\left( n+1 \right)\left( m+1 \right)}^{*} \right)+\left( 1-\delta_{m,0} \right)\mathrm{Im}\left( \alpha_{onm}\alpha_{o\left( n+1 \right)\left( m+1 \right)}^{s*}+\alpha_{onm}^{s}\alpha_{o\left( n+1 \right)\left( m+1 \right)}^{*}+\beta_{onm}\beta_{o\left( n+1 \right)\left( m+1 \right)}^{s*}+\beta_{onm}^{s}\beta_{o\left( n+1 \right)\left( m+1 \right)}^{*} \right) \right\}+C_{nm}^{3}\left\{ \left( 1+\delta_{m,0} \right)\mathrm{Im}\left( \alpha_{enm}\beta_{on\left( m+1 \right)}^{s*}+\alpha_{enm}^{s}\beta_{on\left( m+1 \right)}^{*} \right)+\left( 1-\delta_{m,0} \right)\mathrm{Im}\left( \alpha_{en\left( m+1 \right)}^{s}\beta_{onm}^{*}+\alpha_{en\left( m+1 \right)}\beta_{onm}^{s*} \right) \right\}+C_{nm}^{4}\left\{ \left( 1-\delta_{m,0} \right)\left[ \left( 1-\delta_{m,1} \right)\mathrm{Im}\left( \beta_{enm}^{s}\alpha_{on\left( m-1 \right)}^{*}+\beta_{enm}\alpha_{on\left( m-1 \right)}^{s*} \right)+\left( 1+\delta_{m,1} \right)\mathrm{Im}\left( \beta_{en\left( m-1 \right)}\alpha_{onm}^{s*}+\beta_{en\left( m-1 \right)}^{s}\alpha_{onm}^{*} \right) \right] \right\},$$

$$F_{nm}^{y,ss}={-C}_{nm}^{1}\left\{ \left( 1-\delta_{m,0} \right)\left[ \left( 1-\delta_{m,1} \right)\mathrm{Im}\left( \alpha_{enm}^{s}\alpha_{o\left( n+1 \right)\left( m-1 \right)}^{s*}+\beta_{enm}^{s}\beta_{o\left( n+1 \right)\left( m-1 \right)}^{s*} \right)+\left( 1+\delta_{m,1} \right)\mathrm{Im}\left( \alpha_{onm}^{s*}\alpha_{e\left( n+1 \right)\left( m-1 \right)}^{s}+\beta_{onm}^{s*}\beta_{e\left( n+1 \right)\left( m-1 \right)}^{s} \right) \right] \right\}-C_{nm}^{2}\left\{ \left( 1+\delta_{m,0} \right)\mathrm{Im}\left( \alpha_{enm}^{s}\alpha_{o\left( n+1 \right)\left( m+1 \right)}^{s*}+\beta_{enm}^{s}\beta_{o\left( n+1 \right)\left( m+1 \right)}^{s*} \right)+\left( 1-\delta_{m,0} \right)\mathrm{Im}\left( \alpha_{onm}^{s*}\alpha_{e\left( n+1 \right)\left( m+1 \right)}^{s}+\beta_{onm}^{s*}\beta_{e\left( n+1 \right)\left( m+1 \right)}^{s} \right) \right\}-C_{nm}^{3}\left\{ \left( 1+\delta_{m,0} \right)\mathrm{Im}\left( \alpha_{enm}^{s}\beta_{en\left( m+1 \right)}^{s*} \right)+\left( 1-\delta_{m,0} \right)\mathrm{Im}\left( \alpha_{on\left( m+1 \right)}^{s*}\beta_{onm}^{s} \right) \right\}-C_{nm}^{4}\left\{ \left( 1-\delta_{m,0} \right)\left[ \left( 1+\delta_{m,1} \right)\mathrm{Im}\left( \alpha_{enm}^{s*}\beta_{en(m-1)}^{s} \right)+\left( 1-\delta_{m,1} \right)\left( \beta_{onm}^{s*}\alpha_{on(m-1)}^{s} \right) \right] \right\},$$

$$F_{nm}^{y,si}=-C_{nm}^{1}\left\{ \left( 1-\delta_{m,0} \right)\left[ \left( 1-\delta_{m,1} \right)\mathrm{Im}\left( \alpha_{enm}^{s}\alpha_{o\left( n+1 \right)\left( m-1 \right)}^{*}+\beta_{enm}^{s}\beta_{o\left( n+1 \right)\left( m-1 \right)}^{*}+\alpha_{enm}\alpha_{o\left( n+1 \right)\left( m-1 \right)}^{s*}+\beta_{enm}\beta_{o\left( n+1 \right)\left( m-1 \right)}^{s*} \right)+\left( 1+\delta_{m,1} \right)\mathrm{Im}\left( \alpha_{onm}^{s*}\alpha_{e\left( n+1 \right)\left( m-1 \right)}+\beta_{onm}^{s*}\beta_{e\left( n+1 \right)\left( m-1 \right)}+\alpha_{onm}^{*}\alpha_{e\left( n+1 \right)\left( m-1 \right)}^{s}+\beta_{onm}^{s*}\beta_{e\left( n+1 \right)\left( m-1 \right)}^{s} \right) \right] \right\}-C_{nm}^{2}\left\{ \left( 1+\delta_{m,0} \right)\mathrm{Im}\left( \alpha_{enm}^{s}\alpha_{o\left( n+1 \right)\left( m+1 \right)}^{s*}+\beta_{enm}^{s}\beta_{o\left( n+1 \right)\left( m+1 \right)}^{*}+\alpha_{enm}\alpha_{o\left( n+1 \right)\left( m+1 \right)}^{s*}+\beta_{enm}\beta_{o\left( n+1 \right)\left( m+1 \right)}^{s*} \right)+\left( 1-\delta_{m,0} \right)\mathrm{Im}\left( \alpha_{onm}^{s*}\alpha_{e\left( n+1 \right)\left( m+1 \right)}+\beta_{onm}^{s*}\beta_{e\left( n+1 \right)\left( m+1 \right)}+\alpha_{onm}^{*}\alpha_{e\left( n+1 \right)\left( m+1 \right)}^{s}+\beta_{onm}^{*}\beta_{e\left( n+1 \right)\left( m+1 \right)}^{s} \right) \right\}-C_{nm}^{3}\left\{ \left( 1+\delta_{m,0} \right)\mathrm{Im}\left( \alpha_{enm}\beta_{en\left( m+1 \right)}^{s*}+\alpha_{enm}^{s}\beta_{en\left( m+1 \right)}^{*} \right)+\left( 1-\delta_{m,0} \right)\mathrm{Im}\left( \alpha_{on\left( m+1 \right)}^{s*}\beta_{onm}+\alpha_{on\left( m+1 \right)}^{*}\beta_{onm}^{s} \right) \right\}-C_{nm}^{4}\left\{ \left( 1-\delta_{m,0} \right)\left[ \left( 1+\delta_{m,1} \right)\mathrm{Im}\left( \alpha_{enm}^{s*}\beta_{en\left( m-1 \right)}+\alpha_{enm}^{*}\beta_{en\left( m-1 \right)}^{s} \right)+\left( 1-\delta_{m,1} \right)\mathrm{Im}\left( \beta_{onm}^{s*}\alpha_{on\left( m-1 \right)}+\beta_{onm}^{*}\alpha_{on\left( m-1 \right)}^{s} \right) \right] \right\},$$

$$F_{nm}^{z,ss}=C_{nm}^{5}\left\{ \left( 1+\delta_{m,0} \right)\mathrm{Im}\left( \alpha_{enm}^{s}\alpha_{e(n+1)m}^{s*}+\beta_{enm}^{s}\beta_{e(n+1)m}^{s*} \right)+\left( 1-\delta_{m,0} \right)\mathrm{Im}\left( \alpha_{onm}^{s}\alpha_{o(n+1)m}^{s*}+\beta_{onm}^{s}\beta_{o(n+1)m}^{s*} \right) \right\}+C_{nm}^{6}\left\{ \mathrm{Im}\left( \beta_{enm}^{s}\alpha_{onm}^{s*}+\alpha_{enm}^{s}\beta_{onm}^{s*} \right) \right\},$$

$$F_{nm}^{z,si}=C_{nm}^{5}\left\{ \left( 1+\delta_{m,0} \right)\mathrm{Im}\left( \alpha_{enm}\alpha_{e(n+1)m}^{s*}+\alpha_{enm}^{s}\alpha_{e(n+1)m}^{*}+\beta_{enm}\beta_{e(n+1)m}^{s*}+\beta_{enm}^{s}\beta_{e(n+1)m}^{*} \right)+\left( 1-\delta_{m,0} \right)\mathrm{Im}\left( \alpha_{onm}\alpha_{o(n+1)m}^{s*}+\alpha_{onm}^{s}\alpha_{o(n+1)m}^{*}+\beta_{onm}\beta_{o(n+1)m}^{s*}+\beta_{onm}^{s}\beta_{o(n+1)m}^{*} \right) \right\}+C_{nm}^{6}\left\{ \mathrm{Im}\left( \beta_{enm}\alpha_{onm}^{s*}+\beta_{enm}^{s}\alpha_{onm}^{*}+\alpha_{enm}\beta_{onm}^{s*}+\alpha_{enm}^{s}\beta_{onm}^{*} \right) \right\},$$

$$C_{nm}^{1}=\frac{\left( n+m \right)!}{\left( n-m \right)!}\frac{n\left( n+2 \right)}{\left( 2n+3 \right)\left( 2n+1 \right)},C_{nm}^{2}=\frac{\left( n+m+2 \right)!}{\left( n-m \right)!}\frac{n\left( n+2 \right)}{\left( 2n+3 \right)\left( 2n+1 \right)},C_{nm}^{3}=\frac{\left( n+m+1 \right)!}{\left( n-m-1 \right)!}\frac{1}{\left( 2n+1 \right)},$$

$$C_{nm}^{4}=\frac{\left( n+m \right)!}{\left( n-m \right)!}\frac{1}{\left( 2n+1 \right)},C_{nm}^{5}=\frac{\left( n+m \right)!}{\left( n-m \right)!}\frac{2n\left( n+2 \right)\left( n+m+1 \right)}{\left( 2n+3 \right)\left( 2n+1 \right)},C_{nm}^{6}=\frac{\left( n+m \right)!}{\left( n-m \right)!}\frac{2m}{\left( 2n+1 \right)},$$

$$\alpha_{enm}= p_{nm}+p_{n(-m)}\left[ \left( -1 \right)^{m}\frac{\left( n-m \right)!}{\left( n+m \right)!} \right],\alpha_{onm}=i\left\{ p_{nm}-p_{n(-m)}\left[ \left( -1 \right)^{m}\frac{\left( n-m \right)!}{\left( n+m \right)!} \right] \right\},$$

$$\beta_{enm}=q_{nm}+q_{n(-m)}\left[ \left( -1 \right)^{m}\frac{\left( n-m \right)!}{\left( n+m \right)!} \right],\beta_{onm}= i\left\{ q_{nm}-q_{n(-m)}\left[ \left( -1 \right)^{m}\frac{\left( n-m \right)!}{\left( n+m \right)!} \right] \right\},$$

$$\alpha_{en0}= p_{n0},\alpha_{on0}=0,\beta_{en0}=q_{n0},\beta_{on0}=0,$$

$$\alpha_{enm}^{s}= a_{nm}+a_{n(-m)}\left[ \left( -1 \right)^{m}\frac{\left( n-m \right)!}{\left( n+m \right)!} \right],\alpha_{onm}^{s}=i\left\{ a_{nm}-a_{n(-m)}\left[ \left( -1 \right)^{m}\frac{\left( n-m \right)!}{\left( n+m \right)!} \right] \right\},$$

$$\beta_{enm}^{s}=b_{nm}+b_{n(-m)}\left[ \left( -1 \right)^{m}\frac{\left( n-m \right)!}{\left( n+m \right)!} \right],\beta_{onm}^{s}= i\left\{ b_{nm}-b_{n(-m)}\left[ \left( -1 \right)^{m}\frac{\left( n-m \right)!}{\left( n+m \right)!} \right] \right\},$$

$$\alpha_{en0}^{s}= a_{n0},\alpha_{on0}^{s}=0,\beta_{en0}^{s}=b_{n0},\beta_{on0}^{s}=0.$$

**2. Optical force on a nanoparticle in a nanobubble with 1^st^ scattering approximation**

We use the 1st scattering approximation to estimate the optical force on the nanoparticle when it is a nanobubble. The 1st scattering approximation assumes that an optical force from the reflected fields by the nanobubble interfaces is not dominated [2]. With this approximation, we can efficiently estimate the optical force on a nanoparticle placed at a “certain location” inside a nanobubble. The optical configuration of supercavitating NP is a non-concentric system (i.e., the center of the nanoparticle can be different from that of the nanobubble). To evaluate the optical force in section 1, we need to represent the internal field of nanobubble in the spherical coordinate with the origin at the center of the nanoparticle.

The internal field of the nanobubble ($\mathbf{E}_{\mathbf{n}}$) is from a plane wave that is incident to the nanobubble, and $\mathbf{E}_{\mathbf{n}}$ can be first expressed by VSH in a spherical coordinate that has the origin at the center of the nanobubble:

$$\mathbf{E}_{\mathbf{c}}= \sum_{l=1}^{\infty} \sum_{k=-n}^{n} \left[ d_{lk}\boldsymbol{N'}_{lk}^{\left( 1 \right)}+c_{lk}\boldsymbol{M'}_{lk}^{\left( 1 \right)} \right],$$

where the prime ($\boldsymbol{'}$) on VSH denotes that it has the origin at the center of the nanobubble. As the center of the nanobubble is different from that of the nanoparticle, it is necessary to transform the VSH coefficient of the internal field of the nanobubble ($d_{lk}$ and $c_{lk}$) into those of the nanoparticle. One may have this transformation as the following:

$$p_{nm}= \sum_{l=1}^{\infty} \sum_{k=-n}^{n} \left[ A_{nm}^{lk}(\boldsymbol{r}_{nb\to np})d_{lk}+B_{nm}^{lk}(\boldsymbol{r}_{nb\to np})c_{lk} \right],$$

$$q_{nm}=\sum_{l=1}^{\infty} \sum_{k=-n}^{n} \left[ A_{nm}^{lk}(\boldsymbol{r}_{nb\to np})c_{lk}+B_{nm}^{lk}(\boldsymbol{r}_{nb\to np})d_{lk} \right].$$

In the equation, $A_{nm}^{lk}$ and $B_{nm}^{lk}$ are the complex coefficients for the coordinate transformation from the center of nanobubble to that of the NP ($\boldsymbol{r}_{nb\to np}$) [3]. We note that in the reference [3], the first kind of spherical Hankel function in equation (54) should be replaced with the spherical Bessel function as the internal field of the cavity has a finite value at the center.

In the meantime, the scattered field for the nanoparticle can be obtained by Mie theory as the following:

$$a_{nm}=-\frac{{(n}_{n}/n_{b})\psi_{n}^{'}\left( {\left( \frac{2\pi}{\lambda_{0}} \right)n_{b}R_{n}} \right)\psi_{n}\left( \left( \frac{2\pi}{\lambda_{0}} \right)n_{n}R_{n} \right)-\psi_{n}\left( \left( \frac{2\pi}{\lambda_{0}} \right)n_{b}R_{n} \right){\psi^{'}}_{n}\left( \left( \frac{2\pi}{\lambda_{0}} \right)n_{n}R_{n} \right)}{{(n}_{n}/n_{b})\xi_{n}^{'}\left( \left( \frac{2\pi}{\lambda_{0}} \right)n_{b}R_{n} \right)\psi_{n}\left( \left( \frac{2\pi}{\lambda_{0}} \right)n_{n}R_{n} \right)-\xi_{n}\left( \left( \frac{2\pi}{\lambda_{0}} \right)n_{b}R_{n} \right){\psi^{'}}_{n}\left( \left( \frac{2\pi}{\lambda_{0}} \right)n_{n}R_{n} \right)}p_{nm},$$

$$b_{nm}=-\frac{\psi_{n}^{'}\left( \left( \frac{2\pi}{\lambda_{0}} \right)n_{c}R_{n} \right)\psi_{n}\left( \left( \frac{2\pi}{\lambda_{0}} \right)n_{n}R_{n} \right)-{(n}_{n}/n_{b})\psi_{n}\left( \left( \frac{2\pi}{\lambda_{0}} \right)n_{b}R_{n} \right){\psi^{'}}_{n}\left( \left( \frac{2\pi}{\lambda_{0}} \right)n_{n}R_{n} \right)}{\xi_{n}^{'}\left( \left( \frac{2\pi}{\lambda_{0}} \right)n_{b}R_{n} \right)\psi_{n}\left( \left( \frac{2\pi}{\lambda_{0}} \right)n_{n}R_{n} \right)-{{(n}_{n}/n_{b})\xi}_{n}\left( \left( \frac{2\pi}{\lambda_{0}} \right)n_{b}R_{n} \right){\psi^{'}}_{n}\left( \left( \frac{2\pi}{\lambda_{0}} \right)n_{n}R_{n} \right)}q_{nm},$$

where $\psi_{n}$ and $\xi_{n}$ are Ricatti-Bessel functions, and the prime denotes the differentiation, $\lambda_{0}$ is the vacuum wavelength of the incident plane wave, $n_{n}$ is the complex refractive index of nanoparticle, $n_{b}$ is the refractive of nanobubble, $R_{n}$ is the radius of nanoparticle.

**3. Absorption quality factor of a single nanoparticle**

The absorption quality factor of single nanoparticle facing a plane wave in a water can be evaluated as [3]:

$$Q_{a}=\frac{1}{R_{n}^{2}} \frac{4}{\left( \left( \frac{2\pi n_{w}}{\lambda_{0}} \right) \right)\left| \frac{n_{n}}{n_{w}} \right|^{2}} \times$$

$$\mathrm{Re}\sum_{n=1}^{\infty} \sum_{m=-n}^{n} \frac{i\left( n\left( n+1 \right)\left( n+m \right)! \right)}{\left( 2n+1 \right)\left( n-m \right)!}\left[ {\psi^{'}}_{n}\left( \left( \frac{2\pi}{\lambda_{0}} \right)n_{n}R_{n} \right){\psi^{*}}_{n}\left( \left( \frac{2\pi}{\lambda_{0}} \right)n_{n}R_{n} \right) \right]\left[ \left( \frac{n_{n}}{n_{w}} \right)^{*}\left| e_{nm} \right|^{2}+\left( \frac{n_{n}}{n_{w}} \right)\left| f_{nm} \right|^{2} \right],$$

where

$$e_{nm}=-\frac{i\left( \frac{n_{n}}{n_{w}} \right)}{{(n}_{n}/n_{w})\xi_{n}^{'}\left( \left( \frac{2\pi}{\lambda_{0}} \right)n_{w}R_{n} \right)\psi_{n}\left( \left( \frac{2\pi}{\lambda_{0}} \right)n_{n}R_{n} \right)-\xi_{n}\left( \left( \frac{2\pi}{\lambda_{0}} \right)n_{w}R_{n} \right){\psi^{'}}_{n}\left( \left( \frac{2\pi}{\lambda_{0}} \right)n_{n}R_{n} \right)}{p^{'}}_{nm},$$

$$f_{nm}=-\frac{i\left( \frac{n_{n}}{n_{w}} \right)}{\xi_{n}^{'}\left( \left( \frac{2\pi}{\lambda_{0}} \right)n_{w}R_{n} \right)\psi_{n}\left( \left( \frac{2\pi}{\lambda_{0}} \right)n_{n}R_{n} \right)-{{(n}_{n}/n_{w})\xi}_{n}\left( \left( \frac{2\pi}{\lambda_{0}} \right)n_{w}R_{n} \right){\psi^{'}}_{n}\left( \left( \frac{2\pi}{\lambda_{0}} \right)n_{n}R_{n} \right)}{q^{'}}_{nm},$$

$${p^{'}}_{n1}=-\frac{\left( \frac{1}{2} \right)\left( i \right)^{n+1}\left( 2n+1 \right)}{n\left( n+1 \right)} , {p^{'}}_{n,-1}=\left( \frac{1}{2} \right)\left( i \right)^{n+1}\left( 2n+1 \right),$$

$${q^{'}}_{n1}={p^{'}}_{n1}, {q^{'}}_{n,-1}={-p^{'}}_{n,-1}, {p^{'}}_{nm}= {q^{'}}_{nm}=0 for |m|\neq1,$$

and $n_{w}$ is the refractive index of water.

**4. Absorption quality factor and the sweet zone of a single Au nanoparticle**

**
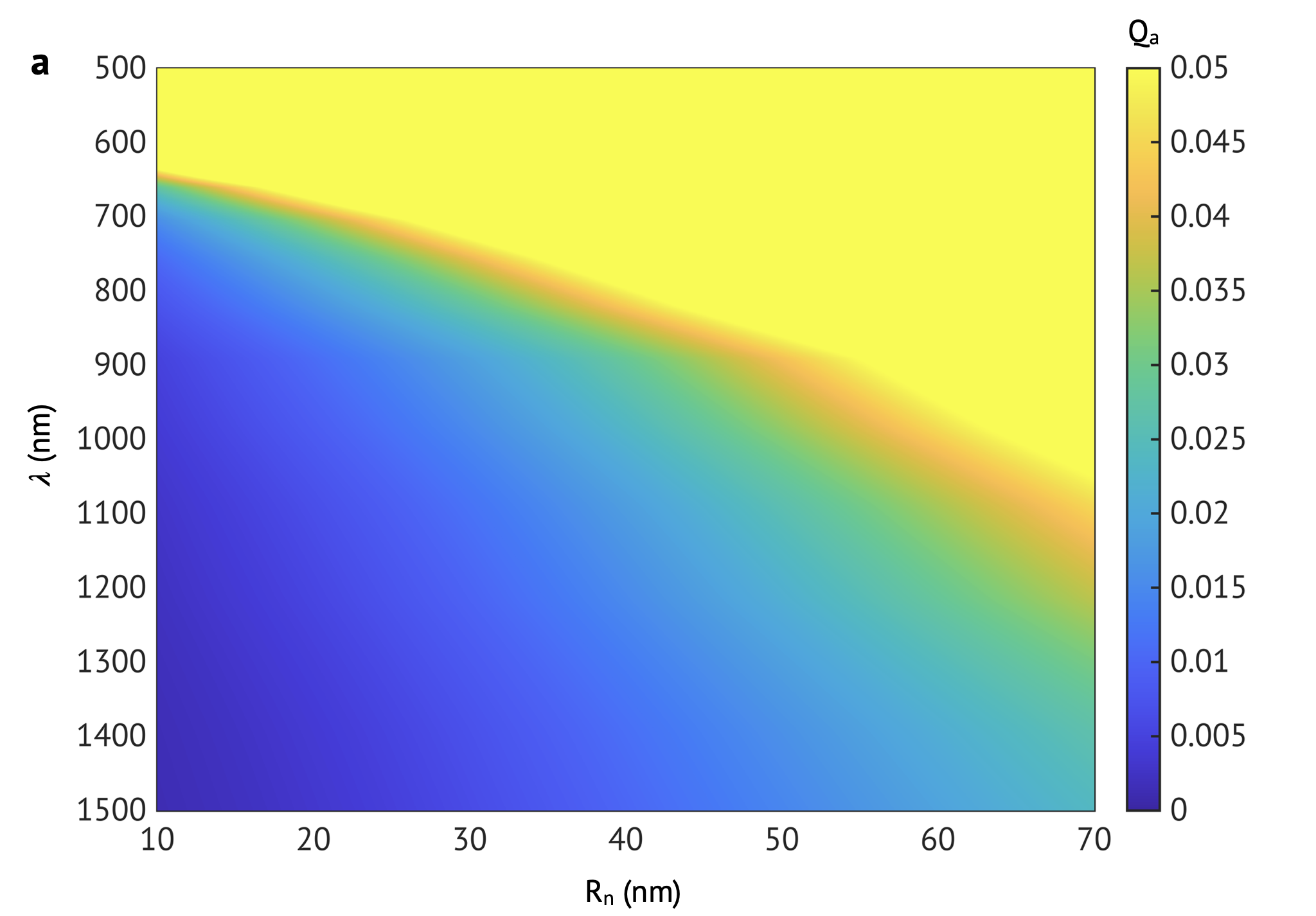
**

**
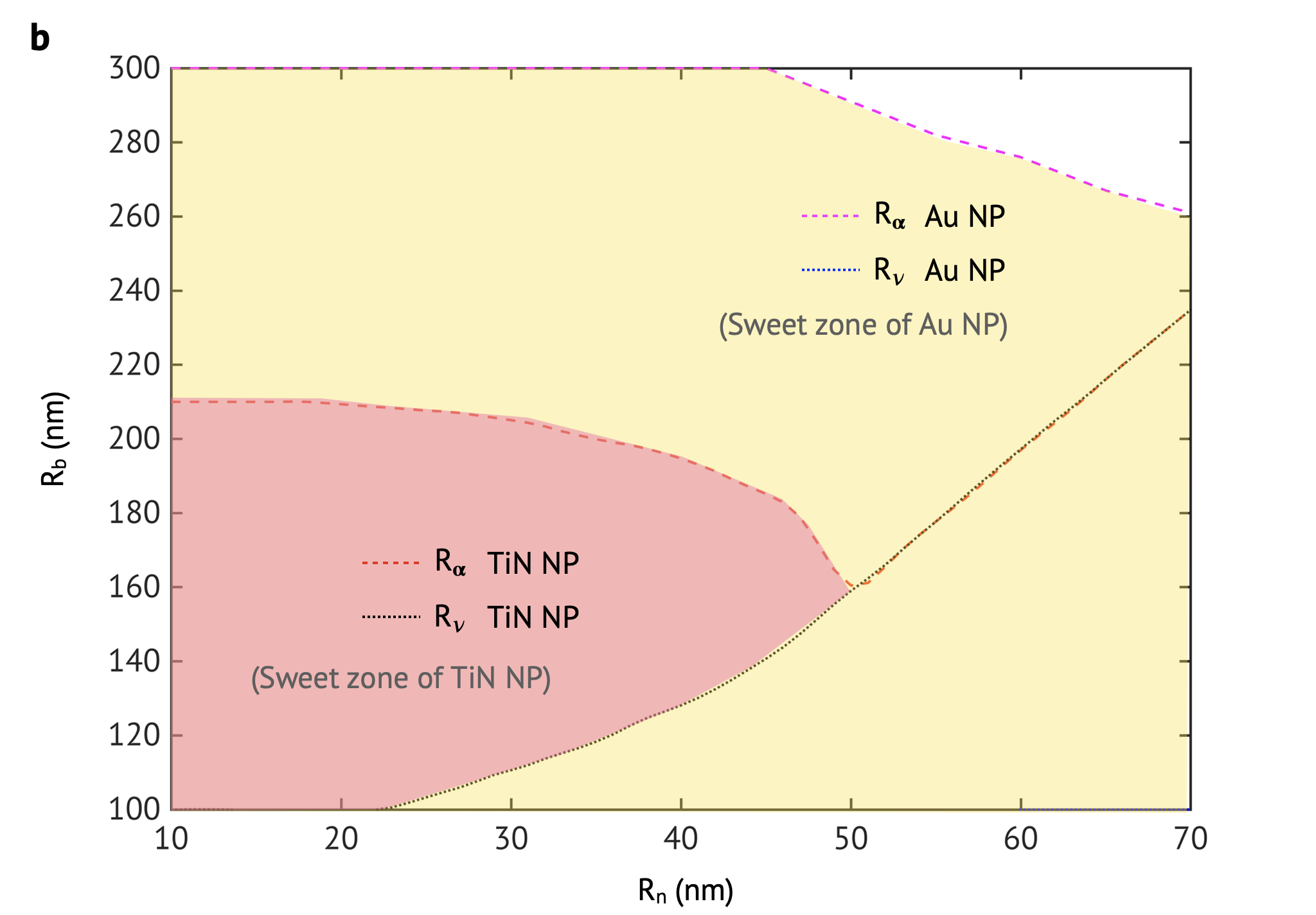
**

**Figure S1** (a) The calculated absorption quality factor of a single Au nanoparticle in water (b) The sweet zone (highlighted in yellow) of negative optical force on a single Au nanoparticle in a nanobubble in water. Note that the sweet zone of a TiN nanoparticle (highlighted in pink) is also shown for comparison.

References

1. A. Salandrino, S. Fardad, and D. N. Christodoulides, "Generalized Mie theory of optical forces," J. Opt. Soc. Am. B **29**, 855 (2012).

2. E. Lee and T. Luo, "Long-distance optical pulling of nanoparticle in a low index cavity using a single plane wave," Sci. Adv. **6**, eaaz3646 (2020).

3. D. W. Mackowski, "Analysis of Radiative Scattering for Multiple Sphere Configurations," Proc. R. Soc. A Math. Phys. Eng. Sci. **433**, 599–614 (2006).
